# Effects of Long COVID on Psycho-Physical Conditions in the Italian Population: A Statistical and Large Language Model Combined Description

**DOI:** 10.3390/ejihpe14050076

**Published:** 2024-04-27

**Authors:** Roberto Lupo, Elsa Vitale, Ludovica Panzanaro, Alessia Lezzi, Pierluigi Lezzi, Stefano Botti, Ivan Rubbi, Maicol Carvello, Antonino Calabrò, Alessandra Puglia, Luana Conte, Giorgio De Nunzio

**Affiliations:** 1 “San Giuseppe da Copertino” Hospital, ASL (Local Health Authority) of Lecce, 73043 Copertino, LE, Italy; roberto.lupo@uniba.it; 2 Department of Mental Health, ASL (Local Health Authority) of Bari, 70100 Bari, BA, Italy; vitaleelsa@libero.it; 3 C.R.A.P. Carrubo, Sol Levante Srl, 74020 Avetrana, TA, Italy; ludovica.panzanaro@hotmail.it; 4 ANT Italian Onlus Foundation (National Cancer Association), 73100 Lecce, LE, Italy; 1alessia.lezzi@gmail.com; 5 “Veris Delli Ponti” Hospital, ASL (Local Health Authority) of Lecce, 73020 Scorrano, LE, Italy; pierlezzi@gmail.com; 6 Hematology Unit, Azienda USL-IRCCS of Reggio Emilia, 42100 Reggio Emilia, RE, Italy; stefano.botti@ausl.re.it; 7 School of Nursing, University of Bologna, 40126 Bologna, BO, Italy; 8 Community Hospital, ASL (Local Health Authority) of Romagna, 48121 Ravenna, RA, Italy; maicol.carvello2@unibo.it; 9 “Nuovo Ospedale Degli Infermi” Hospital, ASL (Local Health Authority), 13900 Biella, BI, Italy; anto.cala76@gmail.com; 10 Perrino Hospital, ASL (Local Health Authority) of Brindisi, 72100 Brindisi, BR, Italy; alessandra.puglia@live.it; 11 Laboratory of Biomedical Physics and Environment, Department of Mathematics and Physics “E. De Giorgi”, University of Salento, 73100 Lecce, LE, Italy; giorgio.denunzio@unisalento.it; 12 Advanced Data Analysis in Medicine (ADAM), Laboratory of Interdisciplinary Research Applied to Medicine (DReAM), ASL (Local Health Authority) and University of Salento, 73100 Lecce, LE, Italy

**Keywords:** long COVID, SARS-CoV-2, generative artificial intelligence, large language models, GPT, LangChain

## Abstract

Background: Long COVID refers to the persistence or development of signs and symptoms well after the acute phase of COVID-19. Objective of the study: To investigate the long-term outcomes of the SARS-CoV-2 infection in terms of psychological, social, and relational consequences within the Italian population. Materials and methods: We conducted an observational, cross-sectional, and multicenter study using an online questionnaire distributed to a sample of the Italian population. By utilizing the Short Form 12 Health Survey (SF-12) and the Hikikomori scale, we assessed perceived quality of life and social isolation, respectively. The questionnaire also included an open-answer question: “What will you remember about the pandemic period?”. We used generative artificial intelligence to analyze and summarize the corresponding answers. Results: A total of 1097 people participated in this study. A total of 79.3% (n = 870) of participants declared that they had been hospitalized and 62.8% (n = 689) received home care. Physical symptoms included headaches (43%, n = 472) and asthma (30.4%, n = 334). Additionally, 29.2% (n = 320) developed an addiction during the pandemic and, among these, 224 claimed internet addiction while 73 declared an emotional addiction. Furthermore, 51.8% (n = 568) experienced limitations in carrying out daily life activities. According to the Hikikomori scale, participants with positive SARS-CoV-2 infection exhibited higher levels of isolation compared to the others (*p* < 0.001). Participants without COVID-19 showed higher levels of emotional support (*p* < 0.001). Our semiautomatic analysis of the open-ended responses, obtained by a procedure based on a free large language model, allowed us to deduce and summarize the main feelings expressed by the interviewees regarding the pandemic. Conclusions: The data collected emphasize the urgent need to investigate the consequences of long COVID in order to implement interventions to support psychological well-being.

## 1. Introduction

Globally, at the time of writing, there have been more than 762.2 million COVID-19 confirmed cases, of which more than 6.8 million people died [1]. The rapid transmission of the COVID-19-related respiratory infection inevitably triggered a common fear of being infected, along with the scarcity of personal protective equipment (PPE), especially in the early days of the pandemic. Added to this, there was the initial lack of a vaccine and treatment approaches; the reduced number of hospital beds and machinery (such as, for example, mechanical respirators); and containment measures such as social distancing and the use of masks, as well as the various lockdowns, followed by great misinformation on this issue [2,3].

The COVID-19 pandemic has had consequences not only on the physical health but also on the psychological well-being of the entire population [2]. Additionally, many people who did not contract the disease faced strong psychological discomforts, such as staying in crowded places for a long time and having close contact with other cohabitants. Those who contracted the infection often perceived posttraumatic stress disorder, as found, for example, in the Norwegian, English, American, and Australian adult populations [3]. According to the criteria of the World Health Organization (WHO), “the post COVID-19 condition occurs in subjects with a positive history of probable or confirmed SARS-CoV-2 infection, generally 3 months after the previous acute condition with symptoms lasting at least for 2 months and which cannot be explained by alternative diagnoses” [4]. Long COVID patients experience several typologies of signs and symptoms which can continue or reappear after the COVID-19 condition. Therefore, healthcare organizations should plan several clinical pathways to face these new, consequential requirements [5]. Individuals of all ages and with all degrees of severity of the acute infection can suffer from long COVID, which generally alters patients’ everyday life [2,3,4]. The literature refers to “long COVID”, also known as “post-COVID syndrome”, as a condition comprising a protracted course of various physical and neuropsychiatric symptoms that persist for more than 12 weeks with no alternative explanation and no known etiology [6]. Its symptoms include persistent fatigue, tiredness and shortness of breath, weakness, muscle and joint pain, lack of appetite, and high blood pressure. More specifically, symptoms include air hunger (often dyspnea), persistent cough, tachycardia, recurring headache, concentration and memory difficulties (the so-called mental fog or “brain fog”); impairment of smell, taste, or hearing, nausea, vomiting, loss of appetite, abdominal pain, diarrhea, gastroesophageal reflux, sleep disturbances, depressed mood (sadness, irritability, impatience, lack of interest in previously enjoyed activities), anxiety, stress, depression, and psychosis [7]. The NICE guidelines [8] propose a representative schematic classification as follows: acute COVID-19 (symptoms up to 4 weeks), continuous COVID-19 (symptoms from 4 to 12 weeks), and post-COVID-19 (symptoms developed during or after the infection which continue for more than 12 weeks).

For these reasons, the risk of marginalization of subjects who, due to the manifestations of this long-term disease, can no longer carry out some activities that they performed before [9] is extremely high.

In light of these observations, the purpose of this study was to investigate the consequences of the long COVID syndrome on the psychological, social, and relational well-being of a sample of the Italian population.

## 2. Materials and Methods

### 2.1. Study Design

The study conducted was observational, cross-sectional, and multicenter with a snowball sampling methodology. This study was carried out from April 2022 to February 2023.

### 2.2. Participants

All the Italian individuals aged between 18 and 80 years who spontaneously agreed to participate and gave their explicit consent for participation were involved in the present study.

### 2.3. Data Collection

The questionnaire was administered online, with a short presentation that preceded the connection link to the form, through various social networks and communication apps, such as Facebook and WhatsApp, and by direct email; a QR code specially created was placed in various shops and was also distributed at various medical practices in the province of Lecce. This dissemination mode allowed us to reach all Italian regions.

### 2.4. The Questionnaire

The main data collection tool was a questionnaire made up of 60 items divided into 7 sections. The first part of the questionnaire collected socio-demographic data, including whether the participant had contracted COVID-19 or not. If the participant answered affirmatively, a series of clinical conditions, referred to in the current literature, were asked about, and the participant could answer only “yes” or “no”. Some clinical conditions investigated included hospitalization, mechanical ventilation treatment, and addictions. The second part of the questionnaire contained questions about the perceived quality of life, in accordance with the Short Form 12 Health Survey (SF-12). The SF-12 explores social, family, and work issues and how these dimensions have been changed by the lockdown and the pandemic in general. Subsequently, the Hikikomori scale was administered to assess social isolation through various factors, such as socialization, isolation, and emotional support. A further section of the questionnaire was used to investigate the individual measures adopted, such as using antidepressants and requests for help from family and/or friends and psychologists. The questionnaire concluded with an open-ended question asking the participant to express in a few simple words what memories the participant would bring with them from the pandemic period.

### 2.5. Ethical Considerations

The questionnaire proposed was totally anonymous. Before proceeding with the compilation, the participants signed the informed consent, which expressly inquired about each participant’s willingness to accept to join and also stated the aim of the study proposed.

The questionnaire adhered to the Helsinki principles and was approved by the Ethical committee of the General Hospital of Policlinic of Bari, Italy, with ID number 0040143/02/05/2022.

### 2.6. Statistical Analysis

All data collected were processed with IBM SPSS software, version 20. The data collected, with the exception of the Hikikomori scale, were presented as frequencies and percentages (with respect to the total number of participants), and a chi-square analysis was performed to assess any differences between participants who had suffered from COVID-19 and those who had not. Then, the effect size in chi-square statistics was also assessed, thanks to Cramer’s method, in which associations varied from 0 to 1. A value of 0 indicated no association between variables, while a value of 1 indicated a perfect association. Finally, the Hikikomori scores were presented as continuous variables and then assessed as means ± standard deviations. Finally, ANOVA tests were performed between each subdimension of the Hikikomori scale and participants who suffered or not from COVID-19. All *p*-values < 0.05 were considered as statistically significant.

### 2.7. Generative Artificial Intelligence and Large Language Models for Open-Ended Question Processing

Data processing for questionnaire items with answers consisting of free numeric values or values selected from a limited number of options (multiple-choice questions or rating scales) is easy and well established. In contrast, the problem of extracting information from answers to open-ended questions and drawing useful insights has currently no standard and solid solution. Natural language processing (NLP) methods such as sentiment/emotion analysis [10] give interesting hints on the interviewee’s feelings but are limited to capturing quite rigid aspects of their attitude and often fall short in representing the complex moods expressed by individuals in their writing. Sentiment analytics just uses a positive, neutral, or negative numeric parameter which summarizes the person experience, while emotion detection can assign scores to well-defined emotions, such as fear, rage, happiness.

The recent popularization of artificial intelligence (AI)-driven large language (generative) models (LLMs) [11] has revolutionized the field of NLP, paving the way for unprecedented advancements in understanding the complex nuances of human expression. These powerful models, such as OpenAI’s GPT (generative pretrained transformer) and ChatGPT, have demonstrated a remarkable ability to generate coherent and contextually relevant text, enabling new possibilities in various applications, including capturing the mood of people from written text. Large generative models leverage deep learning architectures trained on massive amounts of preexisting text data to learn the complexities of language and generate contextually appropriate responses. 

One of the applications of LLMs is the generation of concise text summaries. They analyze the input text, identify key ideas, and extract the most salient information to produce a condensed version that captures the essence of the original content. In this context, it is important to remember that LLMs usually face quite stringent limitations in the length of the input text they can effectively process; computational complexity and resource requirements (memory, CPU/GPU load) rapidly grow with text length. For this reason, strategic choices such as splitting text into chunks that are fed one by one to the LLM (with an eye to the need for preserving context between calls) are in order.

We decided to use LLMs for an automatic semiquantitative analysis of the answers to the last (open-ended) question of our questionnaire, which was “What will you remember of the COVID-19 pandemic?” We soon realized that this was a particularly tough task for an LLM because the interviewees’ answers (hereafter referred to as “the (text) items”) were often very short, making text interpretation difficult. 

The specific purpose of the analysis we performed can be synthesized into two steps:

1. Splitting the whole set of items into a small and arbitrary number of clusters in order to capture patterns in the items themselves, with the corresponding numbers of each cluster.
2. For each specific pattern, summarizing the items in the cluster in order to obtain a more detailed description of the mood expressed in the item cluster.

After obtaining the machine elaboration, we manually re-elaborated the results obtained in order to build a cleaner text for publishing. We also reran the procedure to check for substantial consistency. The result was a detailed description of the main moods expressed by the interviewees.

We decided to use freely available LLMs, though this might in principle imply lower quality in pattern capturing and in each cluster’s summarization. After some experimentation with several models available through the HuggingFace (https://huggingface.co/ (accessed on 20 December 2023)) community, the “flan-alpaca-large” model (https://huggingface.co/declare-lab/flan-alpaca-large (accessed on 20 December 2023)) [12] from the DeCLaRe Lab (https://huggingface.co/ (accessed on 20 December 2023)) was chosen, as it could effortlessly manage the Italian text and gave quite coherent results.

The choice of Python as the programming language is justified by the large availability of libraries for data processing. In particular, various software packages exist to help the experimenter in a comfortable usage of LLMs. We used the LangChain (https://python.langchain.com/docs/get_started/introduction.html (accessed on 20 December 2023)) tool, a framework for developing applications powered by LLMs. In particular, LangChain offers off-the-shelf “chains”, i.e., structured assemblies of components for accomplishing specific high-level tasks.

**Figure 1:**
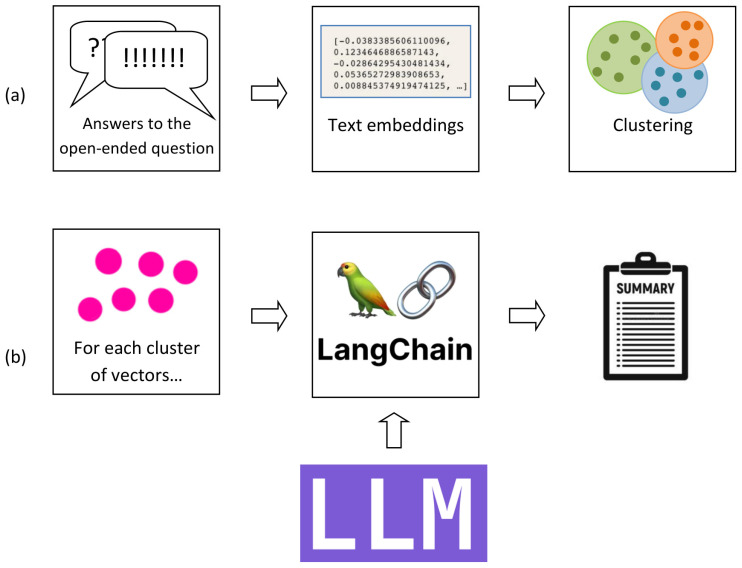
Flowchart of the LLM-based procedure.

Figure 1 shows the flowchart of our procedure. 

Step (a) was performed by first applying vector embedding to all the text items then clustering the obtained set of vectors by the k-means clusterization method. Vector embedding plays an important role in capturing the nature of textual information. It refers to representing words or sentences as vectors in a mathematical space and captures the semantic meaning of what is being embedded. It allows one to search in the dataset by similarity (minimum distance between the search sentence vector and each of the stored vectors), thus making it possible to clusterize the items by semantics following well-known clusterization methods. Various kinds of vector embedding exist, which leaves much space for experimentation and allows one to look for the most effective approach for one’s problem. We used the Hugging Face Hub embedding (characterized by 768 variables) by its LangChain wrapper, which left us with a matrix of 731 × 768 values, 731 being the number of actual non-empty replies to the open-ended question (out of 1106 responders). The embedding vectors were clustered by the k-means algorithm by arbitrarily imposing 8 to 10 clusters as the target partitioning scheme.

## 3. Results

Table 1 collects all the sampling characteristics of the participants.

Sixty-five percent of the sample came from Southern Italy. The most represented gender was female (56.6%), the average age was less than 30 years (46.5%), and the responders were predominantly students (33.9%). A total of 54% of the participants were married, and only 8.8% lived alone.

Table 2 reports different clinical conditions of the participants, differentiating between individuals who had suffered from COVID-19 or not. Significant differences were reported between participants who received home care assistance (*p* < 0.001), as 689 participants declared this condition. However, 763 participants continued to receive their treatments during the COVID-19 outbreak (*p* < 0.001). Significantly high percentages in depression, anxiety, and headache (*p* < 0.001) were reported among participants suffering from COVID-19. The trend seemed to be inverse for the same individuals as regards asthma (*p* < 0.001), gastroesophageal reflux (*p* < 0.001), low back pain (*p* < 0.001), hypertension (*p* < 0.001), menstrual pain (*p* = 0.001), eating disorders (*p* = 0.034), ageusia (*p* < 0.001), regular rest (*p* < 0.001), and others not mentioned above (*p* = 0.024). However, associations between variables were very weak, as shown in Table 2.

Additionally, a significantly high percentage (*p* = 0.003) of the participants (54.5%, computed with respect to the whole sample) declared that they had suffered from COVID-19 but had developed no dependence. However, the effect size was weaker for all the significant differences and in most cases even nonexistent. On the other hand, the other participants, suffering from COVID-19 (29.2% of all the participants) or not (3.8%), declared that they had developed a dependence, especially on internet use (*p* = 0.007).

By considering the subjective perception of quality of life, significant differences were reported among participants (Table 3). Most of those who declared themselves to have suffered from COVID-19 perceived their quality of life as very good (61.7% of all the participants, *p* < 0.001). Additionally, most of the participants who contracted the SARS-CoV-2 infection defined their health conditions to be partially limiting daily activities (51.8% of the whole sample, *p* < 0.001). Finally, the physical and health conditions of COVID patients almost always (25.4% of the sample) or partially (36.9% of all the responders) interfered with their social relationships. However, the effect size for all the significant differences was inexistent (Table 3).

In Table 4, participants’ relations were investigated by highlighting a significant good quality-of-life perception both in individuals suffering and not suffering from COVID-19 (Table 4). However, the effect size was weaker for all the significant differences and in most cases also inexistent (Table 4).

Finally, the condition of social withdrawal was explored in its three related subdimensions, according to COVID-19 and no-COVID-19 participants (Table 5).

Significant differences were reported in the isolation and the emotional support condition (*p* < 0.001), as participants suffering from COVID-19 reported higher levels in the isolation dimension than the others (*p* < 0.001). The trend was inverse as regards the emotional support dimension (*p* < 0.001).

**Figure 2:**
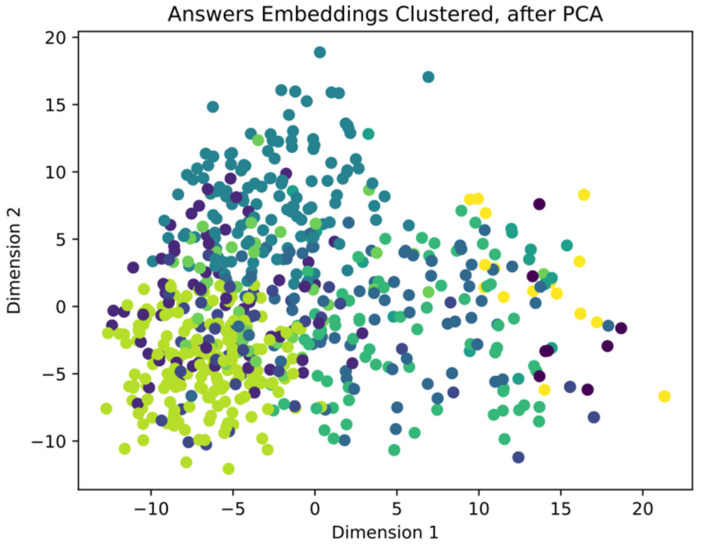
Answers from the open-ended question, coded by vector embedding and after PCA, shown in the two most representative dimensions.

As concerns the semiautomatic analysis of the answers to the open-ended question, Figure 2 shows how the clusterized vectors, calculated from the 768 answers, were placed in a two-coordinate space defined by principal component analysis (PCA). PCA was applied with the only purpose of allowing a simple view of the variable space. The points are colored according to the obtained clusters. 

The clusters do not look neatly defined and separated one from the other for at least two reasons. First of all, it is reasonable that short text items like the given answers to the questionnaire (usually composed of a few words) cannot express sharp concepts with well-defined semantics, so their embedding vectors will easily be spread in the vector space without clear boundaries between groups. Secondly, the PCA representation only shows two dimensions which express a small overall variance (about 13%), so other components are equally important but are not represented in the 2D graph, which only shows projections in a two-dimensional space, with apparent cluster mixing. 

The cardinality of each cluster varied from about 20 to 200 text items each. The partially stochastic nature of k-means clustering makes the result only partly stable and reproducible, so we reran the procedure many times to assess the substantial coherence between runs. 

In step (b) of the procedure, for each cluster, we filtered and included all and only the text items belonging to it and summarized them by the LLM. We used the LangChain “stuffing” method. This is a way to summarize text by feeding a whole document to an LLM in a single call, which can be faster than other methods that require multiple calls and may result in a better summary as the LLM has access to all the data at once. LLMs have a context length, which is the maximum number of tokens that can be processed in a single call. If the document is longer than the context length, the stuffing method will not work and other approaches can help, such as MapReduce and Refine. These approaches can give better performance to improve latency and scalability, at the expense of a certain loss of information. In our case, we did not exceed the toke limit. The temperature of the LLM was fixed to 0 to make the model deterministic and so avoiding further variability.

Visual inspection of the text items of each cluster showed that a certain internal consistency actually characterized each group, though internal variability was also significant, and sometimes a cluster might be split into two different patterns.

**Figure 3:**
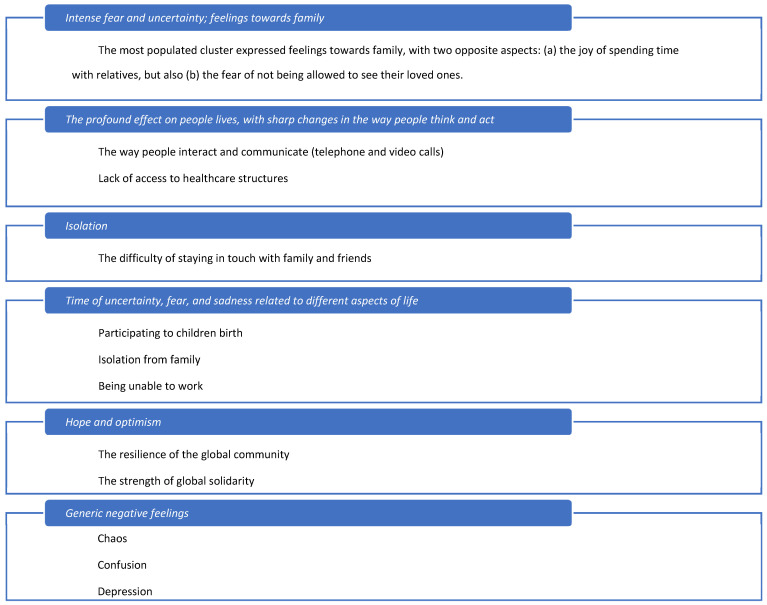
Summary of the answers given to the open-ended question “What will you remember about this pandemic period?”

The result of summarizing the single clusters is shown in Figure 3, where the order top to bottom is from the most to the least populated cluster. Manual intervention was necessary to avoid repetitions and to homogenize the text. Also, some defects in the translation from Italian to English were present, with a few Italian words used in the LLM answers. Clusters suffered from a certain semantic overlap, which was partly manually removed to simplify the scheme.

Of course, the method is not free from defects and needs a good amount of manual intervention, but it may be of help in situations where a large number of answers to open-ended questions in a questionnaire forbids one from visually reviewing all the interviewees’ answers for manual pattern recognition and information deduction.

## 4. Discussion

The purpose of this study was to investigate the outcomes of the COVID-19 condition and especially the long COVID syndrome on the physical, psychological, social, and relational spheres of the Italian population.

The long COVID condition has been recognized as a full-fledged syndrome, as a set of symptoms and clinical signs that constitute the manifestations of one or several diseases. In July 2022, the long COVID cases were assessed around 50 million globally, a number which to date has inevitably and inexorably risen almost a year after the estimation [13,14]. In fact, several studies showed that around 10–20% of the infected people could continue to develop diagnosable symptoms such as long COVID [15]. Long COVID became a presence, sometimes silent, sometimes deafening, but now omnipresent in the lives of many of us, without us necessarily recognizing it. A total of 1097 Italians, with a female majority (56.6%) and a majority residing in the South of Italy (65%) participated in this study. Almost half of the participants were less than 30 years old (46.5%), and 54.1% of them were married; only 44.3% of the total had children. A large portion had obtained a secondary school diploma (n = 445). The majority of the sample was made up of students (n = 372). As regards the most common manifestations following the infection, they were anxiety (60.2% of participants had COVID-19 and suffered from anxiety), depression (47.4%), headache (43%), and asthma (30.4%). In addition to these, 224 participants declared they had developed an addiction. 

There were several stressors that pushed many to an immoderate use of technological devices up to addiction, such as financial, social, health, and work factors developing into gambling, online shopping, and gaming. These pathological behaviors could be interpreted as a coping strategy towards a life radically changed in all its aspects due to the restrictions but also to distract oneself from feelings such as fear, anxiety, and concern that during the pandemic, and the first lockdown in particular, have been omnipresent in our lives [16,17,18]. In fact, the COVID-19 pandemic, in addition to being cited as the main factor in the increase in internet use, has been recognized as an increasing factor in psychiatric diseases [19,20,21]. Furthermore, according to another study which recognized internet use as problematic, this level of internet use could very likely be associated with the severity of wanting to avoid at all costs facing real-life situations related to COVID-19 and the pandemic [22]. 

Many studies reported an increase in time spent on internet-related activities (gaming, social networks, smartphones) during the quarantine compared to that spent before the pandemic. A report on addictions (2019–2021) in Germany, Switzerland, Japan, and the United Kingdom showed that the pandemic created a real addiction and a consequential increasing number of requests for helping conditions, too [23,24,25,26,27]. The I-PACE proposed model (interaction of person–affect–cognition–execution model) showed that some subjects began to have problematic behaviors regarding internet use by adopting various online activities to deal with their psychological distress [28]. Consequently, a vicious circle was established with internet excessive usage in order not to face their own problems. This unhealthy use became the cause of further conflicts and a reason for stress, further fueling the use of the internet as a coping strategy. This addiction seemed to be “normalized” by the fact of seeing oneself surrounded by other people, such as family members or friends, who used the internet as much, so that this condition almost acquired the characteristics of normality, thus passing in an extremely dangerous way as socially acceptable [29]. The results of our study show that 51.8% of participants who tested positive for COVID-19 were currently limited in their daily life activities due to the previously contracted infection and that 36.9% (n = 405) of participants felt that their own emotions interfered in their social and relational activities. Moreover, a sense of abandonment (23.4%) and loneliness (24.5%), as well as discomfort in crowded places (34.1%), increased in those who contracted COVID-19.

The participants also filled in the section concerning the Hikikomori scale, which allowed them to evaluate their condition regarding socialization, isolation, and emotional support. From this section, it emerged that participants who contracted COVID-19 reported higher levels in the isolation dimension than the other two dimensions investigated, while those who did not contract COVID-19, were more vulnerable in terms of emotional support. Unfortunately, there are still not many empirical studies on the matter given the young age of the phenomenon in relation to the pandemic, but this syndrome was found in Brazil and reported in three different studies [29,30,31,32]. According to some researchers, the regime of self-isolation might have caused the manifestation of some markers of Hikikomori in subjects prone to the practice of isolation and in those with psychological preconditions, such as, for example, the desire not to leave one’s room even when the opportunity came [33]. When forced isolation was imposed by various governments, sometimes just a little more than legality reasons was given to these individuals to avoid contact with the outside world. 

During the COVID-19 pandemic, young people began to show a willingness to withdraw into the home, especially those with a low level of emotional intelligence, leading them into a vortex of profound existential solitude [34]. Craparo et al. [35] reported a significant association between the long COVID condition and dysfunctional personality traits, such as negative affectivity, detachment, antagonism, disinhibition, and psychoticism. Therefore, the COVID-19 pandemic has been a catalyst for the Hikikomori phenomenon both in people who already showed, even subconsciously, the will to isolate themselves from the rest of the world and in those who, before the advent of COVID-19, had no apparent desire for isolation. This yet more proof of the serious effects the years after the disease have had on the psychological, social, and relational well-being and balance of the global population in general and the Italian one in particular, according to this study. What has emerged is a society that is frail, lonely, and unprepared. This has been demonstrated by the loneliness of many people confined to their homes with no escape and the unpreparedness of our hospitals in responding to such a massive emergency. 

Going into the details of this study, data showed an increase in depression, anxiety, agitation in crowded places, and feelings of loneliness and abandonment [36]. The physical and psychological components in the long COVID syndrome intersected, with one often becoming the consequence of the other. In fact, lack of social stimuli and activities in healthy individuals and patients with cognitive disabilities seems to have favored the onset of anxiety and depression, with subsequent cognitive decline and impairment [37]. There is a high probability that psychiatric symptoms, neurological and physical illnesses, and inflammatory brain damage in individuals with post-COVID-19 syndrome increased suicidal ideation and behaviors. Depression, anxiety disorders, PTSD, sleep disturbances, and cognitive impairments were associated with suicidal behavior [37,38]. The increase in internet addiction cases moreover should certainly be counted among the most relevant results of this study even though this did not come as a surprise, since the internet has been, during the various lockdowns, the only window to the external world in a world that no one was recognizing anymore. 

## 5. Limitations

This study underlines how little known long COVID still is and how much it is necessary to study this particular disease to deepen our knowledge especially in the future when the effects, both physical and psychological, will be evaluated in the very long distance. However, the short amount of time between the COVID-19 pandemic and the present time represents a limit as well. Other limitations are due to the fact that the questionnaire was administered online, making it available only for those who had access to the internet. Last to be included in the list of limitations are selection and recall bias. 

## 6. Conclusions

The main objective of this study was to analyze the effects of the long COVID syndrome, the lockdowns, and the pandemic not only on the single individual but on the individual as an integral part of the whole society. The research also helped us to understand the bright sides of this dark period.

Long COVID represents a constant reminder of who we were before, who we were in between, and who we have become. This virus has reminded us how weak humanity is but above all how equal it is: the virus hit with no distinction of gender, race, or sexual or political orientation. From this work and others, the urgency emerges to intervene with promptness to help persons showing emotional and material hardships as a consequence of the virus. This study highlighted the loneliness of many people confined to their homes with no escape route and the unpreparedness of our hospitals in responding to such an emergency. Going into the details of this study, data showed an increase in depression, anxiety, agitation in crowded places, and feelings of loneliness and abandonment, confirmed by the expressions used by individuals to define this terrible circumstance: “intense fear and uncertainty, as well as the joy of being able to share their lives with others, in particular with family and friends; the fear of being unable to see their loved ones; the difficulty of coping with the loss of jobs, education, and even their own lives; a profound effect on people’s lives, related to profound changes in the way people think and act; a time of fear and isolation; the resilience of the global community, chaos, confusion, depression, anxiety”. This study finally shows how fundamental it is to promote psychological well-being both of individuals and of the community through meetings, group activities, and, above all, follow-up programs for those who are the new chronic sufferers of this pretty new disease.

## Figures and Tables

**Table 1:** Sampling characteristics of the participants (n = 1097).

Sampling Characteristics	n (%)
Region of Italy	
North	216 (19.7)
Center	168 (15.3)
South	713 (65)
Gender	
Female	621 (56.6)
Male	476 (43.4)
Age	
Up to 30 years	510 (46.5)
31–40 years	174 (15.9)
41–50 years	136 (12.4)
51–60 years	124 (11.3)
Over 60 years	153 (13.9)
Marital status	
Married	594 (54.1)
Unmarried	456 (41.6)
Divorced/separated	26 (2.4)
Widower/widow	21 (1.9)
Educational level	
Lower	214 (19.5)
Diploma	445 (40.6)
Degree	272 (24.8)
Postdegree	78 (7.1)
None	88 (8)
Occupational level	
Student	372 (33.9)
Housewife	87 (7.9)
Worker	116 (10.6)
Public employee	179 (16.3)
Freelance	163 (14.9)
Retired	110 (10)
Unemployed	32 (2.9)
Other	38 (3.5)
Children	
Yes	486 (44.3)
No	611 (55.7)
Live with	
Family of origin	358 (32.6)
Own family	338 (30.8)
Others	304 (27.7)
Alone	97 (8.8)

**Table 2:** Disease perception and treatments during the COVID-19 outbreak. Parentheses report percentages computed with respect to all the respondents.

Item	COVID-19		*p*-Value	Effect Size
	Yes	No		
Were you hospitalized?			0.348	0.288
Yes	48 (4.4)	6 (0.5)		
No	870 (79.3)	173 (15.8)		
Were you on mechanical ventilation during your hospitalization?			0.409	0.255
Yes	23 (2.1)	2 (0.2)		
No	895 (81.6)	177 (16.1)		
Were you subjected to home care assistance?	689 (62.8) 229 (20.9)	14 (1.3) 165 (15)	<0.001 ***	0.000
Yes				
No				
Did you interrupt your treatment due to COVID-19?			<0.001 ***	0.000
Yes	155 (14.1)	6 (0.5)		
No	763 (69.9)	173 (15.8)		
During this time, you suffered from:				
Depression			<0.001 ***	0.000
Yes	520 (47.4)	22 (2)		
No	398 (36.3)	157 (14.3)		
Anxiety			<0.001 ***	0.000
Yes	660 (60.2)	14 (1.3)		
No	258 (23.5)	165 (15)		
Headache			<0.001 ***	0.000
Yes	472 (43)	15 (1.4)		
No	446 (40.7)	164 (14.9)		
Asthma			<0.001 ***	0.000
Yes	334 (30.4)	8 (0.7)		
No	584 (53.2)	171 (15.6)		
Gastroesophageal reflux			<0.001 ***	0.000
Yes	135 (12.3)	5 (0.5)		
No	783 (71.4)	174 (15.9)		
Low back pain			<0.001 ***	0.000
Yes	190 (17.3)	6 (0.5)		
No	728 (66.4)	173 (15.8)		
Hypertension			<0.001 ***	0.000
Yes	143 (13)	3 (0.3)		
No	775 (70.6)	176 (16)		
Menstrual pain			0.001 ***	0.003
Yes	72 (6.6)	3 (0.3)		
No	846 (77.1)	176 (16)		
Eating disorders			0.034 *	0.035
Yes	41 (3.7)	2 (0.2)		
No	877 (79.9)	177 (16.1)		
Ageusia			0.001***	0.003
Yes	72 (6.6)	3 (0.3)		
No	846 (77.1)	176 (16)		
Regular rest			<0.001 ***	0.000
Yes	92 (8.4)	3 (0.3)		
No	826 (75.3)	176 (16)		
Other			0.024 *	0.022
Yes	45 (4.1)	2 (0.2)		
No	873 (79.6)	177 (16.1)		
Have you developed an addiction?			0.003 **	0.003
Yes	320 (29.2)	42 (3.8)		
No	598 (54.5)	137 (12.5)		
What addiction?			0.007 **	0.007
Narcotic substances	5 (0.5)	3 (0.3)		
Alcohol abuse	5 (0.5)	2 (0.2)		
Medicines	6 (0.5)	1 (0.1)		
Gambling	7 (0.6)	2 (0.2)		
Internet	224 (20.4)	24 (2.2)		
Affectivity	73 (6.7)	8 (0.7)		
No addiction	598 (54.5)	139 (12.7)		

Statistical significance at * *p* ≤ 0.05; ** *p* ≤ 0.01; *** *p* ≤ 0.001.

**Table 3:** Quality-of-life perception. Parentheses report percentages, computed with respect to all the respondents.

Item	COVID-19		*p*-Value	Effect Size
	Yes	No		
Overall, you would say your health is			<0.001 ***	0.000
Excellent	27 (2.5)	20 (1.8)		
Very good	677 (61.7)	87 (7.9)		
Good	172 (15.7)	53 (4.8)		
Passable	36 (3.3)	19 (1.7)		
Poor	6 (0.5)	0 (0)		
Your health restricts you from carrying out daily activities			<0.001 ***	0.000
Yes, it limits me a lot	128 (11.7)	6 (0.5)		
Yes, it partially limits me	568 (51.8)	38 (3.5)		
No, it doesn’t limit me at all	222 (20.2)	135 (12.3)		
Has your physical health or emotional state interfered with your social activities, family, friends?			<0.001 ***	0.000
Always	9 (0.8)	5 (0.5)		
Almost always	279 (25.4)	48 (4.4)		
Part of the time	405 (36.9)	61 (5.6)		
Almost never	187 (17)	41 (3.7)		
Never	38 (3.5)	24 (2.2)		

Statistical significance at *** *p* ≤ 0.001.

**Table 4:** Effects of the COVID-19 pandemic on participants’ relationships, health, and economic conditions. Parentheses report percentages, computed with respect to all the respondents.

Items	COVID-19		*p*-Value	Effect Size
	Yes	No		
Relationships with partner			<0.001 **	0.000
In no way	793 (72.3)	114 (10.4)		
Very little	34 (3.1)	30 (2.7)		
A bit	56 (5.1)	19 (1.7)		
Much	22 (2)	13 (1.2)		
Very much	13 (1.2)	3 (0.3)		
Relationships with children			0.009 *	0.000
In no way	870 (79.3)	160 (14.6)		
Very little	21 (1.9)	12 (1.1)		
A bit	15 (1.4)	5 (0.5)		
Much	11 (1)	1 (0.1)		
Very much	1 (0.1)	1 (0.1)		
Relationships with parents			<0.001 **	0.017
In no way	770 (70.2)	107 (9.8)		
Very little	62 (5.7)	35 (3.2)		
A bit	59 (5.4)	26 (2.4)		
Much	22 (2)	7 (0.6)		
Very much	5 (0.5)	4 (0.4)		
Relationships with co-workers			<0.001 **	0.000
In no way	795 (72.5)	129 (11.8)		
Very little	60 (5.5)	22 (2)		
A bit	43 (3.9)	17 (1.5)		
Much	15 (1.4)	10 (0.9)		
Very much	5 (0.5)	1 (0.1)		
Relationships with educational institutions			<0.001 **	0.000
In no way	793 (72.3)	115 (10.5)		
Very little	33 (3)	16 (1.5)		
A bit	48 (4.4)	20 (1.8)		
Much	27 (2.5)	17 (1.5)		
Very much	17 (1.5)	11 (1.0)		
Concentration at school			<0.001 **	0.000
In no way	720 (65.6)	110 (10)		
Very little	59 (5.4)	17 (1.5)		
A bit	64 (5.8)	22 (2)		
Much	48 (4.4)	22 (2)		
Very much	27 (2.5)	8 (0.7)		
Concentration at work environments			0.011 *	0.000
In no way	682 (62.2)	112 (10.2)		
Very little	117 (10.7)	30 (2.7)		
A bit	76 (6.9)	22 (2)		
Much	31 (2.8)	13 (1.2)		
Very much	12 (1.1)	2 (0.2)		
Health condition			0.001 **	0.000
In no way	110 (10)	71 (6.5)		
Very little	525 (47.9)	67 (6.1)		
A bit	222 (20.2)	25 (2.3)		
Much	55 (5)	15 (1.4)		
Very much	6 (0.5)	1 (0.1)		
Sleep disorder			0.001 **	0.000
In no way	422 (38.5)	84 (7.7)		
Very little	196 (17.9)	35 (3.2)		
A bit	238 (21.7)	36 (3.3)		
Much	48 (4.4)	12 (1.1)		
Very much	14 (1.3)	12 (1.1)		
The feeling of abandonment			<0.001 **	0.000
In no way	212 (19.3)	69 (6.3)		
Very little	251 (22.9)	44 (4)		
A bit	334 (30.4)	42 (3.8)		
Much	101 (9.2)	14 (1.3)		
Very much	20 (1.8)	10 (0.9)		
Shaking hands in crowded places			<0.001 **	0.003
In no way	115 (10.5)	42 (3.8)		
Very little	241 (22)	37 (3.4)		
A bit	374 (34.1)	53 (4.8)		
Much	144 (13.1)	23 (2.1)		
Very much	44 (4)	24 (2.2)		
Interest in participating in social and cultural events			0.001 **	0.035
In no way	130 (11.9)	42 (3.8)		
Very little	310 (28.3)	38 (3.5)		
A bit	257 (23.4)	48 (4.4)		
Much	162 (14.8)	32 (2.9)		
Very much	59 (5.4)	19 (1.7)		
Economic condition			0.0998	0.003
In no way	238 (21.7)	62 (5.7)		
Very little	337 (30.7)	53 (4.8)		
A bit	230 (21)	39 (3.6)		
Much	93 (8.5)	19 (1.7)		
Very much	20 (1.8)	6 (0.5)		
The feeling of loneliness			<0.001 **	0.000
In no way	102 (9.3)	47 (4.3)		
Very little	255 (23.2)	41 (3.7)		
A bit	269 (24.5)	36 (3.3)		
Much	194 (17.7)	29 (2.6)		
Very much	98 (8.9)	26 (2.4)		

Statistical significance at * *p* ≤ 0.05; ** *p* ≤ 0.001.

**Table 5:** Hikikomori symptoms and related subdimensions.

Hikikomori Scale/COVID-19	µ ± s.d.	C.I. 95%		F	*p*-Value
		Minimum	Maximum		
Socialization					
COVID-19 yes	19.57 ± 4.17	19.30	19.84	3.50	0.062
COVID-19 no	20.26 ± 5.78	19.40	21.11		
Isolation					
COVID-19 yes	13.94 ± 4.21	13.67	14.21	22.59	<0.001 ***
COVID-19 no	12.18 ± 5.84	11.32	13.05		
Emotional support					
COVID-19 yes	11.35 ± 2.51	11.19	11.51	13.51	<0.001 ***
COVID-19 no	12.12 ± 2.87	11.70	12.55		
Total score					
COVID-19 yes	44.86 ± 8.39	44.32	45.41	0.162	0.687
COVID-19 no	44.56 ± 11.97	42.80	42.80		

Statistical significance at *** *p* ≤ 0.001.

## Data Availability

Data will be available upon request.
